# Recent Advances in Plant-Mediated Zinc Oxide Nanoparticles with Their Significant Biomedical Properties

**DOI:** 10.3390/bioengineering9100541

**Published:** 2022-10-11

**Authors:** Muhanad Alhujaily, Salim Albukhaty, Mohammad Yusuf, Mustafa K. A. Mohammed, Ghassan M. Sulaiman, Hassan Al-Karagoly, Amal A. Alyamani, Jawaher Albaqami, Faizah A. AlMalki

**Affiliations:** 1 Department of Medical Laboratory Sciences, College of Applied Medical Sciences, University of Bisha, Bisha 67714, Saudi Arabia; 2 Department of Chemistry, College of Science, University of Misan, Maysan 62001, Iraq; 3 College of Medicine, University of Warith Al-Anbiyaa, Karbala 56001, Iraq; 4 Department of Clinical Pharmacy, College of Pharmacy, Taif University, Haweiah, Taif 21944, Saudi Arabia; 5 Department of Medical Physics, Al-Mustaqbal University College, Hillah 51001, Iraq; 6 Division of Biotechnology, Department of Applied Sciences, University of Technology, Baghdad 10066, Iraq; 7 Department of Internal and Preventive Medicine, College of Veterinary Medicine, University of Al-Qadisiyah, Al-Diwaniyah 58002, Iraq; 8 Department of Biotechnology, College of Science, Taif University, Taif 21944, Saudi Arabia; 9 Department of Biology, College of Science, Taif University, P.O. Box 11099, Taif 21944, Saudi Arabia

**Keywords:** nanotechnology, green synthesis, zinc oxide nanoparticles, antimicrobial, anticancer, antioxidant

## Abstract

Compared to traditional physical and chemical approaches, nanobiotechnology and plant-based green synthesis procedures offer significant advantages, as well as having a greater range of medical and biotechnological applications. Nanoparticles of zinc oxide (ZnO NPs) have recently been recognized as a promising option for many industries, including optics, electrics, packaged foods, and medicine, due to their biocompatibility, low cytotoxicity, and cost-effectiveness. Several studies have shown that zinc ions are important in triggering cell apoptosis by promoting the generation of reactive oxygen species (ROSs) and releasing zinc ions (Zn^2+^), which are toxic to cells. The toxic nature of the chemicals used in the synthesis of ZnO nanoparticles limits their clinical utility. An overview of recent developments in green ZnO NP synthesis is presented in this review, emphasizing plant parts as reducing agents and their medical applications, including their antimicrobial, anticancer, antioxidant, and anti-inflammatory properties, as well as key mechanisms of action for these applications to facilitate further research on the biomedical fields in the future.

## 1. Introduction

**Figure 1:**
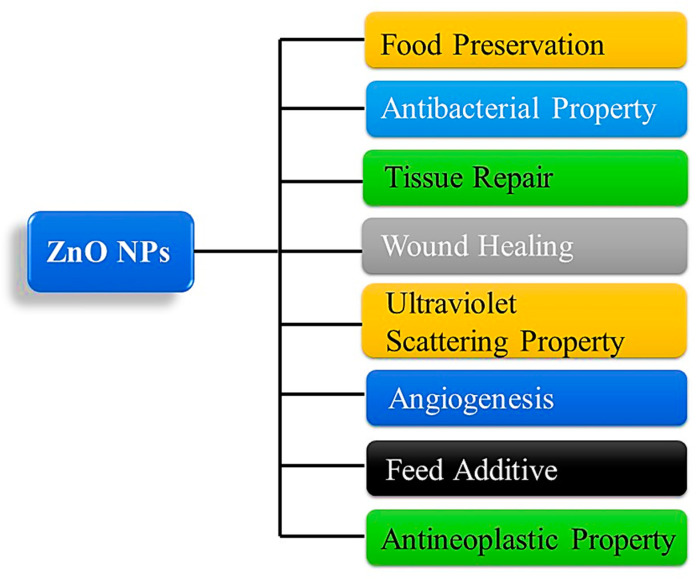
Schematic diagram of common applications of ZnO NP. ZnO, zinc oxide; NPs, nanoparticles.

Nanotechnology is a branch of science and technology that deals with synthesizing and manipulating materials over a very small scale (1–100 nm) [1]. In addition to being used in pharmaceuticals and biomedical fields, it can also be used in the synthesis of nanoparticles for the delivery of drugs, treatment of cancer, diagnosis, and the prevention of infection [2,3,4,5,6]. The high surface area-to-volume ratio of these nanomaterials sets them apart from bulk substances with similar compositions in terms of their physicochemical properties [7]. One of the reasons for nanoparticles’ widespread use in biomedicine is their ability to interact well with biological membranes, receptors, nucleic, and protein acids due to their small size (nanoscale) [8]. The biocompatibility, stability, and safety of ZnO nanoparticles make them an excellent choice for use in medical applications [9]. The Food and Drug Administration (FDA) has approved ZnO-NPs as a pharmaceutical excipient, and it is widely used in prescription formulas and cosmetics [10]. A further property of ZnO is that it has a -OH group that permits it to dissolve at a slow rate in acidic (e.g., cancer cells and tumor microenvironments) as well as strong basic environments, offering the potential to be used in biomedical applications [11]. The use of zinc oxide nanoparticles is widespread in biomedicine against bacteria, viruses, fungi, and parasites, anticancer drugs, and therapeutic agents. Furthermore, zinc oxide is used in the manufacture of concrete, photocatalysis, electronics, electrotechnology, and many other industrial applications [12,13,14,15,16,17,18,19] as shown in Figure 1.

## 2. Traditional Method of Producing NPs

**Figure 2:**
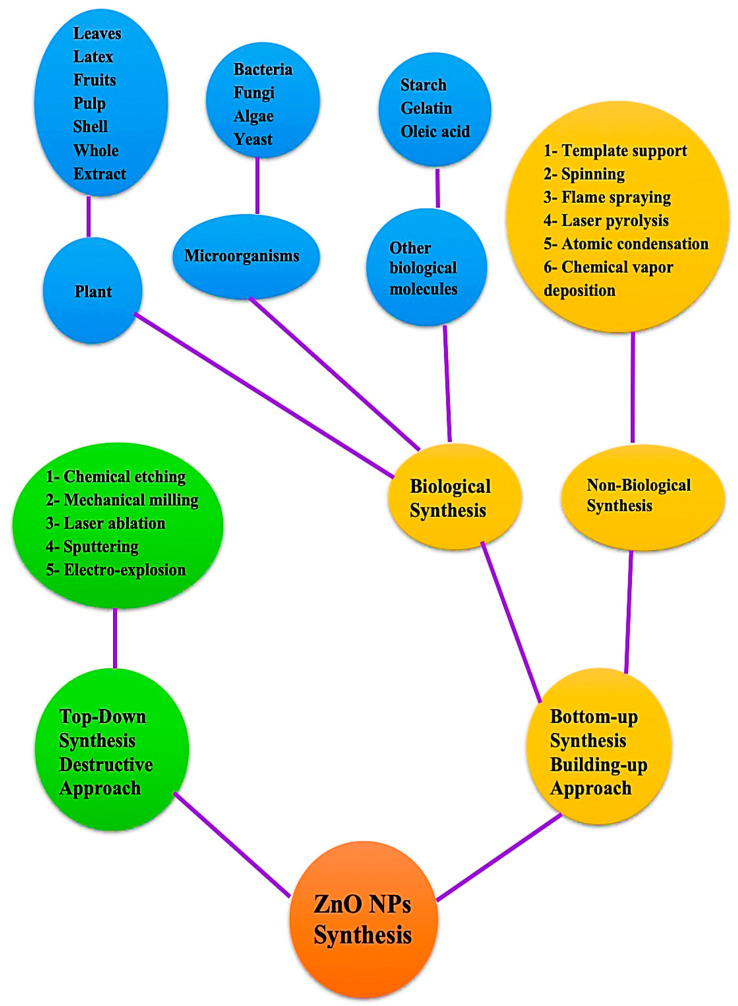
General methods for creating zinc oxide nanoparticles.

Synthesis of NPs can generally be accomplished through a variety of strategies that can be classified as top-down or bottom-up methods. A top-down involves the decomposition of larger molecules into smaller ones, followed by the conversion of these smaller molecules into suitable nanoparticles, this is in contrast to a bottom-up approach in which atomic-sized particles are assembled to produce nanoparticles [20]. In order to manufacture ZnO nanoparticles, several chemical and physical procedures have been employed, such as hydrothermal processes, sol-methods, chemical vapor deposition, precipitations, laser ablations, and physical vapor depositions [21,22,23,24,25,26]. It is important to keep in mind that these methods typically involve the use of organic solvents and hazardous reducing agents, which are in most cases highly reactive and toxic to the environment. In a green synthesis process, microbes and plants are used to create nanoparticles for biomedical applications [27,28,29]. In addition to being environmentally friendly, this process extends the life of nanoparticles, overcoming the limitations of traditional chemical and physical methods for NP synthesis [30]. There are several functional groups in plant phytoconstituents, such as hydroxyl, carboxyl, and amine, that react with metal ions to reduce their size to a nanometer and it is believed that the -OH group in flavonoids is responsible for the reduction of metal ions into NPs. Furthermore, these compounds not only facilitate the biological reduction of ions to nanoscale sizes, but also facilitate the capping of nanoparticles, which are necessary for the stability and biocompatibility of nanoparticles [31,32,33]. The top-down approach can be implemented in several ways such as laser ablation, chemical etching, sputtering, mechanical milling, and so on [34]. In addition to biological techniques, non-biological techniques are also included in the bottom-up strategy, such as spinning, laser pyrolysis, flame spraying, and atomic condensation have been mentioned. Biological strategies are sometimes referred to as green strategies and utilize a variety of biotic resources, including plants, algae, microbes, and other biological components, such as egg albumin, starch, and gelatin, to produce diverse types of nanoparticles [35,36,37,38]. This process used to produce ZnO NPs is safe, environmentally friendly, biocompatible, and simple. Several techniques for producing NP are depicted in Figure 2. The green synthesis approach has grown in significance over the past few years due to its many advantages, including ease of scaling up for large-scale synthesis, low cost, good stability of the nanoparticles produced, and non-toxic byproducts. This makes it the best method for the production of metal oxide nanoparticles such as ZnO NPs compared to other chemical synthesis methods that result in toxic chemical species adsorbed on the surface of nanoparticles. Thus, this study primarily focuses on environmentally sustainable ways to produce ZnO NPs, particularly from plant components, as well as their biomedical applications.

## 3. Green Synthesis of Nanomaterials

**Figure 3:**
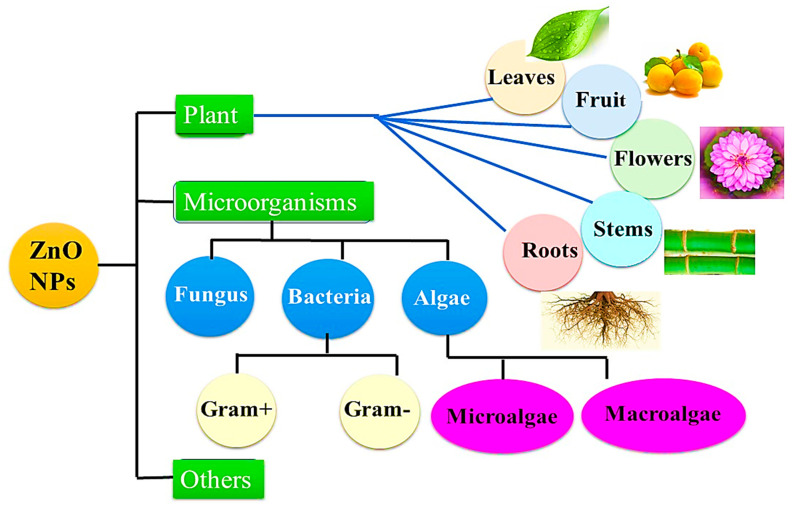
The typical sources of biosynthesis processes used to produce zinc oxide nanoparticles.

A biosynthesis of nanomaterials is a technique that uses plants and biopolymers to form nanoparticles with a wide range of biomedical applications. It is an expense and environmentally friendly approach because harmful chemicals are not present or a large amount of energy is not required, which explains why this innovative approach is so popular. Further benefit of this process is that the pristine substances are naturally rich in amino, carboxyl, and hydroxyl groups, which are commonly utilized as stabilizing agents or capping agents in aqueous solutions, which lead to the formation of nanoparticles [39,40]. A wide range of plants, bacteria, algae, fungi, as well as other biological components, such as starch and egg, have been employed in the biosynthesis of ZnO NPs, as shown in Figure 3. It is well known that natural compounds, especially phytochemicals, found in plant parts, such as leaves, fruit peels, flowers, and seeds were used in the green synthesis of metal oxide NPs. The plant method has many advantages over the microbe approach because it doesn’t need separate, complicated, or many processes, such as isolation, culture development, and maintenance [41]. The extracted solution may be directly used to synthesize ZnO or may be dried to concentrate solid extracts. Following this, various pH and temperature conditions are applied to react zinc precursors and plant extracts [42]. It is necessary to add zinc precursors to the solution if the extract is used as an aqueous solution [43]. Alternatively, the zinc precursor is mixed with distilled water along with the leaf extract powder [44]. The main mechanism of action is the oxidation and reduction of zinc ions by a phytochemical found in natural extracts [45]. As a result of their considerable antimicrobial activity, photodegradation, and metal ion adsorption process, green synthesized ZnO NPs are being used in many fields including biomedicine and biotechnology [46,47,48]. According to current research investigations, nanoparticles synthesized via the green pathway are more effective at inhibiting bacterial growth because they are coated with functional groups derived from phytochemicals [49]. The green synthesis method also exhibits greater catalytic activity and reduces exposure to and use of hazardous and expensive chemicals, which can help to protect the environment from their toxicity. Throughout this review, we will describe some of the applications of ZnO NPs manufactured from natural extracts. 

### Plant-Based Synthesis of ZnO NPs

Researchers have recently given a lot of attention to plant-based nanoparticle synthesis since it is rapid, inexpensive, and environmentally safe. In relatively recent work, Nazir, Arif, et al. investigated the effect of *Rumex dentatus* leaf extract on the formation of ZnO nanoparticles using zinc nitrate precursors, and then successfully used it as an efficient antibacterial agent [50]. Zinc oxide (ZnO) nanoparticles (NPs) were created utilizing thyme leaf extract using a green technique, according to ST Karam et al. The formation of the nanoparticles were spherical in shape with an average size in the range of 39.4–51.86 nm [51]. In a study conducted by Droepenu et al., *Anacardium occidentale* leaf extract was used to produce ZnO NPs with two precursors of zinc salt (zinc acetate dihydrate and zinc chloride) and evaluated against *Acinetobacter baumannii*, *Escherichia coli*, *Staphylococcus aureus*, and *Exiguobacterium aquaticum* [52]. Zinc nitrate hexahydrate was used as a precursor to producing ZnO nanoparticles from the *Vitex negundo* plant, which demonstrated bacterial inhibition against pathogenic gram-positive and gram negative bacteria [53]. According to Aldalbahi et al., zinc nitrate hexahydrate was used as a precursor in the green synthesis of ZnO NPs using an aqueous extract of *K. blossfeldiana*, and its cytotoxicity and anticancer properties were evaluated [54]. *Sambucus ebulus* leaf extract was used to generate zinc oxide nanoparticles (ZnO NPs), which were then investigated for a number of potential future applications [55]. There is evidence to suggest that green-generated ZnO NPs utilizing *Passiflora caerulea* leaf extract may be used to treat urinary tract infections since they are multifunctional inorganic nanoparticles. The obtained ZnO nanoparticles were tested against a pathogenic culture that was extracted from the urine of a patient with a UTI [56]. According to a study, *Parkia roxburghii* seeds extract used to produce ZnO nanoparticles demonstrated outstanding methylene blue (MB) and Rhodamine B dye degradation, reaching nearly 98% [57]. ZnO NPs isolated from extracts of *Allium sativum*, *Rosmarinus officinalis*, and *Ocimum basilicum* have reportedly shown significant antioxidant activity [58]. Recent studies suggest that the synthetic zinc oxide nanoparticles (ZnO NPs), which were produced using zinc nitrate and an aqueous peel extract from *Lagenaria siceraria* (*L. siceraria*), represent an important environmentally friendly alternative to combat malaria parasites and vectors [59]. Examined the performance of ZnO NPs created by *Eucalyptus globulus* as photocatalysts for. Furthermore, Zinc nitrate and *Aloe vera* leaf extract were used to produce stable and spherical ZnO nanoparticles. The formed ZnO NPs were examined for their various properties employing UV-Vis spectrophotometers, FTIR, photoluminescence, XRD, FE-SEM, and TEM [60]. There is no doubt that to produce green nanoparticles using metallic ions, plants are most often used as the substrate [61]. In addition, plant parts such as leaves, stems, roots, fruits, and seeds produce a variety of phytochemicals that have been used to synthesize ZnO NPs, which have been used in a variety of potential applications shown in Table 1.

**Figure 4:**
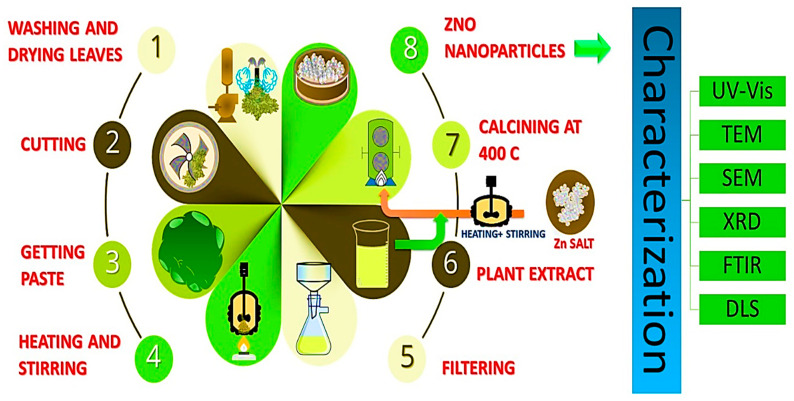
Key steps of the green synthesis of zinc oxide nanoparticles.

In theory, one possible reason for this is that other biological sources are perceived as being more dangerous and more complex than vegetable substrates. Furthermore, plants have the potential for large-scale production and the ability to produce NPs of various shapes and sizes. A precise proportion of plant extract is added with zinc precursors such as zinc nitrate, zinc acetate, or zinc chloride. Following the mixing of the ingredients, Whatman paper was used to achieve a transparent solution that would be used as an extract in the next step. ZnO NPs are subsequently produced by calcining the mixture at a higher temperature. Visual confirmation of the ZnO NPs was achieved by using a color change, while further confirmation was obtained using UV-Vis spectroscopy. The key steps of the green synthesis of zinc oxide nanoparticles are shown in Figure 4. Shows the key steps of the green synthesis of zinc oxide nanoparticles. It is recognized that plant extracts are used in a green synthesis method to create ZnO NPs an exceptional antibacterial effectiveness against a wider variety of germs than is seen with chemically produced. They are therefore non-toxic and gentle on the skin. appropriate for use in products meant for contact with an animal or human. Given these characteristics, synthesis of green ZnO NP using plant extracts can also be used to give fabrics antimicrobial characteristics, while preserving the development of environmental sensitivity [85].

## 4. Mechanism of “Green” Synthesis for ZnO NPs

In a mechanism based on plants leaf extract to synthesize nanoparticles, the extract is incorporated with metal precursors at various conditions to facilitate the synthesis of nanoparticles [86]. A variety of factors related to the conditions of the leaf extract are acknowledged to influence the rate of nanoparticle formation, its yield, and its stability including kinds of phytochemicals, metal salt concentrations, pH, and temperature [87,88,89]. The phytochemicals included in plant extracts have a remarkable ability to decrease metal ions in a much shorter time than fungi and bacteria, which need a longer incubation duration [90]. It has thus been found that plant leaf extracts are benign sources for the biosynthesis of metal oxide NPs as well as metal ions. Moreover, plant leaf extracts function in the NP synthesis process as both reducing and stabilizing agents to stimulate the production of NPs [91]. Additionally, the plant extract’s composition must be taken into account because different plant extracts contain varying levels of phytochemicals [92,93]. Plants contain an array of phytochemicals such as terpenoids, aldehydes, sugars, flavones, ketones, and amides, which contribute to the bioreduction of NPs [94,95,96]. There are various functional groups present in flavonoids that enhance their capacity to reduce metal ions. When flavonoids are converted from their enol form into their keto form, reactive hydrogen atom is released. Metal ions are converted into metal nanoparticles during this process. The enol-to-keto-transformation is responsible for the production of biogenic silver nanoparticles from sweet basil extracts (*Ocimum basilicum*) [97]. Sugars that are present in plant extracts, such as glucose and fructose, may also could contribute in the production of metallic NPs [98,99]. An analysis of FTIR spectra of biosynthesized NPs derived from plants revealed that the nascent NPs were repeatedly related to proteins [100]. It is also important to note that amino acids reduce metal ions in various methods. According to Gruen et al. [101], amino acids (namely cysteine, arginine, lysine, and methionine) exhibit excellent affinity for silver ions in solution [102] Recently, Ebrahiminezhad et al., [103]. examined the impact of amino acids on the reduction of magnetic oxide nanoparticles to identify their efficient potential behavior as effective biocompatible coating agents. The biomolecules in plant extracts consist of carbohydrates and proteins, which serve as reductants to assist the production of metallic NPs [104,105]. Further, the amino groups (-NH) in proteins present in the extracts may also play a significant role in the reduction of metal ions. 

**Figure 5:**
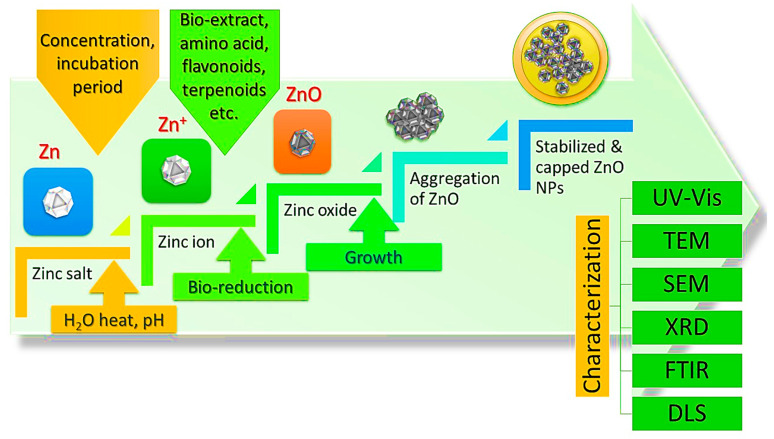
Potential mechanisms in green synthesis of ZnO NPs.

A variety of phytochemicals, such as flavones, alkaloids, phenols, and anthracenes, contain functional groups (such as –C–O–C–, –C–O–, –C=C–, and –C=O–) that could contribute to the generation of metal nanoparticles [106]. In general, liquid capping ligands are primarily responsible for stabilizing NPs to slow-down their further development and agglomeration [107], silver bromide emulsion was used to cover photographic films. As a result of the light shining on the film, the silver bromide was sensitized. Once the film is immersed in a solution of hydroquinone, the silver ion oxidizes the hydroquinone to quinone. During this process, the silver ions were converted into silver metal, which stayed in the emulsion. Considering the chemistry of photography, hydroquinone, plastohydroquinone or quinol (alcoholic compounds) are the major reduction agents in the process of reducing silver ions to silver nanoparticles by non-cyclic photophosphorylation [108]. Therefore, these results showed that biomolecules and heterocyclic chemicals isolated from plant extracts have a role in the extracellular creation of metallic NPs. Recently, it has been shown that several different phytochemicals found in plants can aid in the reduction of metal salt into metallic NPs. These include alkaloids, terpenoids, phenolic acids, sugars, polyphenols, and proteins [109,110] showed, for example, that terpenoids present in geranium leaf extract contribute to nanoparticle formation. There are several terpenoids found in *Cinnamomum zeylanisum* (cinnamon) extracts, including eugenol, which is integral to the bioreduction of the metal salts HAuCl_4_ and AgNO_3_. As a result of FTIR analysis, it was found that the hydroxyl groups (OH) contained in eugenol dissipate during the growth of Au and Ag NPs. It is also observed that functional groups such as carbonyl, alkenes, and chloride are formed after Au NPs being formed. Additionally, other groups, such as R-CH and -OH (aqueous) have also been reported before and after the generation of Au NPs [111]. Based on these findings, the authors suggested the potential chemical mechanism demonstrated in Figure 5. However, the exact mechanism for the preparation of metal oxide nanoparticles using plants remains unclear. It is generally agreed that, metallic nanoparticles are synthesized from plant extracts in three phases: (1) activation (bioreduction of metal ions/salts and nucleation process of the reduced metal ions), (2) growth (spontaneous combination of small particles with larger ones through a process called Ostwald ripening), and (3) termination (determining the final shape of nanoparticles).

## 5. Biomedical Applications

### 5.1. Antimicrobial Properties

**Figure 6:**
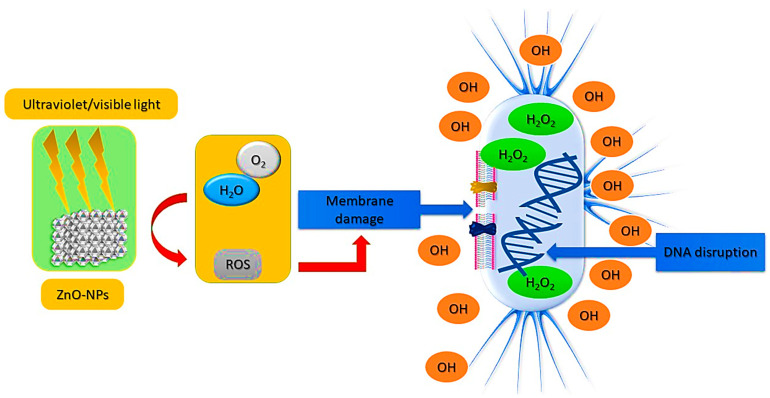
Mechanism of ROS production from ZnO NPs and their bactericidal effect.

Globally, the emergence of antibiotic resistance is one of the most pressing health issues of the 21st century. In this regard, it is necessary to develop an antibiotic agent that is capable of eradicating pathogenic bacteria that have become resistant to medication. Nanoparticles exhibit strong antibacterial properties because they are small and possess a high surface area compared to larger molecules. In addition, nanoparticles penetrate the membrane at varying levels, disrupting it, inserting themselves inside the cells, and inhibiting bacterial protein production [112,113,114]. It has been demonstrated that different metal nanoparticles (NPs), including gold, silver, iron, as well as metal oxide nanoparticles (NPs), such as iron oxide, copper oxide, and cobalt oxide, exhibit antimicrobial properties [115]. ZnO NPs have a potential to act as antibacterial agents, making them of interest to both the biomedical and food industries [116] as presented in Figure 6. Since traditional antibiotics are becoming increasingly resistant to microbial growth, several experiments have been conducted to improve antimicrobial activity. A series of in vitro antimicrobial tests has shown that metallic nanoparticles inhibit a wide range of bacteria species [117]. Metal nanoparticles are characterized by two factors that determine their antimicrobial effectiveness: (i) the materials used in their production; and (ii) the size of their particles. Throughout history, microbial resistance to antibiotics has increased, posing a serious threat to the health of the public. In addition, methicillin-resistant microorganisms can be found in antimicrobial drug-resistant microorganisms. Based on previous investigations show that ZnO NPs produced through green synthesis utilizing plant extracts had inhibitory effects on a range of pathogens, with excellent antimicrobial properties than ZnO NPs made through chemical synthesis. Furthermore, they have significant activity more than antibiotics and bactericides. Consequently, they are optimistic substances that could help in the effort to overcome antibiotics Using medical antibacterial textiles efficiently contributes to sterilizing by reducing the formation of microorganisms, resistance, and bacterial contamination, including antimicrobial bandages, dressings, and gloves.

### 5.2. Antioxidant and Anti-Inflammatory Properties

Oxidative metabolism plays a crucial role in the survival of cells. During this method, free radicals, and ROS are formed, which can lead to certain unintended consequences. It is possible for these free radicals to overpower certain enzymes, including catalase, peroxidase, and superoxide dismutase, and thereby result in lethal impacts on cells via oxidizing proteins, membrane lipids, DNA enzymes, and influencing cell signaling pathways. When excess amounts of these free radicals are produced in the body, cellular respiration ceases. In one hand, oxidation is a critical component of chemical deterioration that can affect food flavor, texture, nutritional value, and safety. On the other hand, antioxidants are available in both natural and synthetic forms to decrease the negative effects of oxidation [110,111]. Additionally, nanoparticles possess powerful antioxidant properties. The leaf extracts of *Sageretia thea* was used to produce plant-mediated ZnO with cubic structures and an average diameter of 20 nm. A variety of antioxidant assays have been performed along with free radical scavenging, antioxidant capacity, and reducing power evaluations. In addition, biogenic ZnO NPs demonstrated superior radical scavenging properties and overall antioxidant capacities above typical values [112]. ZnO NPs were biosynthesized using *Ziziphora clinopodioides* Lam leaves aqueous extract, and their antioxidant properties were evaluated, according to Mahdavi et al. [113]. The results of the green-synthesized nanoparticles were outstanding, as they demonstrated their ability to scavenge DPPH free radicals, ZnO NP concentration increases along with scavenging activity in bioinspired ZnO NPs. It has been shown that ZnO NPs derived from *Sesbania sesban* extract exhibit similar DPPH radical scavenging properties as silver and copper oxide NPs [114].

### 5.3. Anticancer Properties

**Figure 7:**
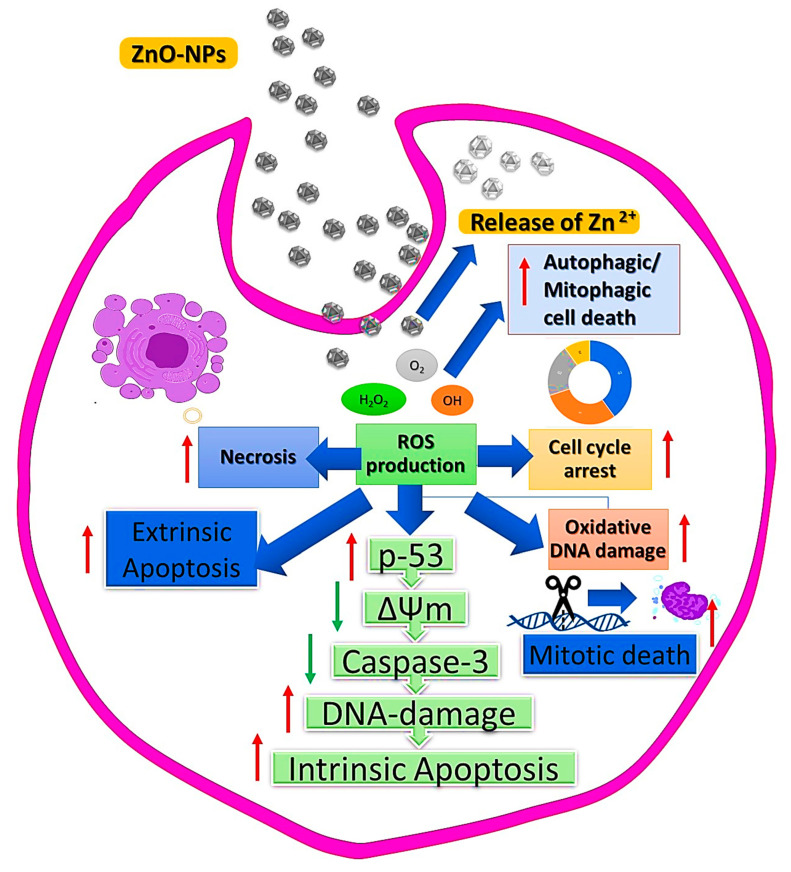
Anticancer mechanism for zinc oxide nanoparticles produced by plant extracts.

It is known that anticancer drugs may damage the mitochondrial electron transport chain and cause significant levels of ROS to develop, since this chain has been implicated in the formation of ROS within cells, as present in Figure 6. Additionally, the presence of excessive ROS will cause mitochondrial damage and impair protein activity, resulting in cell apoptosis [115]. ZnO NPs exhibit some cytotoxicity in cancer cells, primarily due to their ability to release dissolved zinc ions into the cells, cause increased ROS production, and activate the apoptotic signaling pathway [116]. Using human liver cancer cells, HepG2, Sharma et al. [117] evaluated the effects of ZnO NPs and their potential therapeutic mechanisms. It was found that ZnO NPs increased cytotoxicity and genotoxicity in HepG2 cells, which were mediated by ROS-induced mitochondrial dysfunction. When the mitochondrial membrane potential is lost, outer membrane pores open, which lead to the release of apoptotic proteins, such as cytochrome C, into the cytosol and the activation of the caspase enzymes. Moghaddam et al. have biosynthesized ZnO NPs and evaluated their anticancer efficacy in breast cancer MCF-7 cells using a novel yeast strain (Pichia kudriavzevii GY1). ZnO NPs have been observed to exhibit moderate to strong cytotoxicity against MCF-7 cells, with apoptosis being more likely to be induced than cell cycle arrest as the mechanism causing this cytotoxicity [118]. The expression of antiapoptotic genes, such as Bcl-2, AKT1, and JERK/2, was downregulated during apoptosis induced by ZnO NPs, whereas proapoptotic genes, such as p21, p53, JNK, and Bax, were upregulated [119]. A considerable amount of research has been conducted on the use of ZnO NPs for cancer therapy, and it has been shown that they have a specific cytotoxic effect on the growth of cancer cells. According to Chandrasekaran and Pandurangan’s study [120] on the cytotoxicity of ZnO nanoparticles against cultured C2C12 myoblastoma cancer cells and 3T3-L1 adipocytes found that the nanoparticles were more toxic to the cells of C2C12 than to those of 3T3-L1. It was reported that ZnO NPs inhibited proliferation of cancer cells and induced significant amounts of apoptosis through the pathways p53, Bax/Bcl-2 ratio, and caspase-3, as well as mitochondrial intrinsic apoptosis, which is mediated by ROS [121]. According to these findings, ZnO NPs can cause cancer cell death specifically, making them a potential target for cancer therapeutics. Additionally, there is evidence that autophagy and ROS contribute to ZnO NP’s cytotoxicity, however the regulatory mechanisms between autophagy and ROS are not fully understood. A study performed by Zhang et al. [122] investigated autophagy regulation and the relationship between autophagy and ROS in lung epithelial cells treated with ZnO NPs. It appears that ZnO nanoparticles may accumulate autophagosomes in A549 cells and hinder their autophagic flux. When zinc ions were released from ZnO NP in lysosomes, this was positively correlated with the induction of autophagy. Furthermore, the zinc ions released may cause damage to lysosomes and disrupt mitochondrial function which may lead to a build-up of ROS resulting in cell death. With these studies, we gained an improved understanding of the mechanisms governing the autophagy-lysosomes-mitochondria-ROS axis, which will contribute to the development of more effective methods of evaluating the toxicity of nanomaterials. A possible anticancer mechanism for zinc oxide nanoparticles derived from plant extracts is illustrated in Figure 7.

### 5.4. Other Biomedlogical Applications

Apart from their antibacterial, antioxidant, cytotoxic, hemolytic, and antiviral properties, ZnO NPs have many other biological and medicinal applications [123]. It has been reported that cancer was identified as the second most common cause of dysphoria in humans, after cardiovascular diseases [124]. There is evidence that ZnO NPs possess antitumor properties. Accordingly, Aalami et al. produced ZnO nanoparticles from *Saponaria officinalis* extract and examined their anti-proliferative effects on MCF-7, MDA-MB-231, and HFF cells, using the MTT assay [125]. Additionally, several studies have demonstrated that green-synthesized ZnO NPs have demonstrated a high level of wound healing capacity when used as an ointment [126]. It has been demonstrated that biologically produced ZnO NPs have catalytic properties, enzyme inhibition properties, anti-diabetic efficacy, and anticholinergic properties [127,128,129,130]. In addition to its blue and near-UV emitting properties, ZnO NPs also emit green or yellow fluorescence characteristics of oxygen vacancies. Due to their distinct physicochemical features, zinc oxide nanoparticles in the size range of 20–80 nm are frequently used in commercial products such as doping and catalysis. ZnO NPs would influence particular cells’ and tissues’ functionality [131]. ZnO NPs are highly valuable in numerous sectors, including catalysis, gas sensing, electronics, and environmental remediation, and their unique features encourage their incorporation into a variety of commercial products, biotechnology, and biomedical applications [132]. These properties further increase the effectiveness of ZnO NPs for bioimaging applications.

## 6. Conclusions and Future Perspectives

Several properties of these ZnO nanoparticles make them suitable for biomedical applications, including their ability to treat bacteria, inhibit cancer, combat inflammation, deliver drugs, dress wounds, and perform bioimaging. In light of their inherent toxicity, green-synthesized ZnO NPs have the potential to be useful in treating cancer and bacteria by causing ROS to be generated within the cells and activating apoptotic signals. Both bacteria and malignant cells are believed to be inhibited by these ROS. In addition, these manufactured ZnO NPs have long been recognized for their ability to enhance the bioavailability of medicinal medicines or biomolecules when acting as drug carriers to enhance therapeutic efficacy. Generally, the physical or chemical procedures that are used in the fabrication of nanoparticles are detrimental to the environment and require intensive, costly labor. An environmentally friendly method of producing zinc oxide nanoparticles is by utilizing extracts from a wide variety of plants and plant components, as well as biological molecules such as oleic acid, starch, and gelatin. Compared to traditional ZnO NPs, green synthesized ZnO NPs have several advantages including cost-effectiveness, ease of production, environmental safety, and biocompatibility. Through the use of plant components, such as leaf, stem, root, fruit, and seed, we have evaluated current trends and understandings of greenly synthesized ZnO NPs and their medicinal applications in this review. In a related study, researchers described comprehensive scientific data on recent advancements in the methods used to synthesize and characterize ZnO nanoparticles from plant sources [133]. Another study by Akbar et al. gives a summary of numerous investigations on the creation of zinc oxide nanoparticles by plants and their use as antimicrobial agents [134]. On other hand, a similar study conducted by Agarwal et al. [135] included an investigation and characterization techniques used for the environmentally friendly production of ZnO NPs from various biological sources. Although the widespread involvement of polyphenolic compounds in the plant commonwealth could be used to explain how extracts from various plant species are capable of producing NPs, a precise understanding of the green synthesis process is required to realize the full potential of this process in medical and industrial applications. Obtaining homogeneously dispersed NPs is extremely difficult despite the straightforward synthesis of NPs using a green method because many factors, such as temperature, the pH of the system, the type of capping agent used, the concentration of active compounds, etc., may be crucial in determining the size and morphology. It is crucial to compare ZnO NPs produced using medicinal plant extracts to their chemically or physically manufactured equivalents in order to ascertain whether the bioactivities observed may be related to the presence of capping agents in the ZnO NPs.

## Figures and Tables

**Table 1:** A variety of plant parts were utilized to synthesize zinc nanoparticles.

Reducing Factor	Plant Part	Size (nm) and Shape	Characterization	Application	Ref No.
*Hibiscus subdariffa*	Leaf	16–60 nm Spherical	UV, FTIR, XRD, EDX, FESEM, and HRTEM	Anti-diabetic activity	[62]
*Phlomis*	Leaf	79 nm Hexagonal form	UV, FTIR, XRD, EDX, Zeta potential and FESEM	Antibacterial (*S. aureus* and *E. coli*)	[63]
*Aquilegia pubiflora*	Leaf	34.23 nm Elliptical	UV, FTIR, XRD, EDX, Zeta potential and SEM	Anti-Alzheimer	[64]
*Sambucus ebulus*	Leaf	40–45 nm Hexagonal	UV-Vis spectrophotometer, FTIR, XRD, EDX, and FESEM	A photocatalyst for the degradation of dye contaminants	[55]
*Eucalyptus globulus*	Leaf	11.6 nm Spherical	UV, FTIR, XRD, EADX, FESEM, and HRTEM	A ntioxidant activity by DPPH assay	[65]
*Hyssops officinalis L.*	Leaf	20–40 nm Pseudo spherical	UV, FTIR,. FESEM, and DLS	Anti-angiogenesis, anti-inflammatory and cytotoxicity properties	[66]
*Aloe barbadensis miller*	Leaf	25–40nm Spherical	UV, XRD, SEM, and TEM	Optical activity	[67]
*Pomegranate*	Fruit	9.7 ± 3 nm Polyhedral shapes	UV, FTIR, XRD, and HR-TEM	Agent for methylene blue degradation	[68]
*Myristica fragrans*	Fruit	66 nm Elliptical shape	UV-Vis spectrophotometer, XRD, Zeta-potential, TGA, SEM and TEM	Antioxidant activity	[69]
*Sea Buckthorn*	Fruit	17.15 nm Hexagonal	UV, XRD, TGA, XPS and FE-TEM	Improvement of wastewater treatment	[70]
*Ailanthus altissima*	Fruit	5–18 nm Spheres	UV, FTIR, XRD, EDAX, and SEM	Antibacterial against *S. aureus, and E. coli*	[71]
*Rubus ellipticus*	Fruit	20 nm Spherical	UV, FTIR, XRD, TEM, FESEM and XPS	Antioxidant, and antibacterial	[72]
*Syzygium cumini*	Seed	∼48 nm Spherical	UV, FTIR, XRD, and SEM	Water purification	[73]
*Nigella sativa L.*	Seed	20 nm Spherical	UV-Vis spectrophotometer, FTIR, XRD, and SEM	Food additives	[74]
*Foeniculum vulgare Mill*	Seed	23–51 nm Spherical	UV, FTIR, XRD, and TEM	Antimicrobial agent	[75]
*Peganum harmala*	Seed	40 nm None uniform	UV, FTIR, XRD, FESEM, EDX, and TEM	Environmental adsorbent	[76]
*Zingiber officinale*	Root	30–50 nm Spherical	FTIR, XRD, EDX, and SEM	Biological activity	[77]
*Scutellaria baicalensis*	Root	∼40 nm Nearly spherical	UV, FTIR, EDX, FE-TEM, and XRD	Reducing agent for photocatalysis	[78]
*Polygala tenuifolia*	Root	9.22 nm Spherical	UV, FTIR, TEM, SEM and TGA.	anti-inflammatory	[79]
*Sphagneticola trilobata Lin*	Root	65–80nm Spherical	XRD, FTIR, SEM, and EDS	Enhancement plant growth	[80]
*Nyctanthes arbor-tristis*	Flower	12–32 nm Spherical	UV, FTIR, XRD, TEM and DLS	Antioxidant fungal	[81]
Pomegranate (*Punica ranatum*)	Flower	52.50 nm Spike-like	UV, FTIR, XRD, TEM and EDX	Antioxidant bacterial	[82]
*Anchusa italica*	Flower	8–14 nm Spherical	UV, FTIR, TEM, and XRD	Antibacterial and cytotoxicity	[83]
*Trifolium pratense*	Flower	100 nm Spherical	UV, XRD, FTIR EDX and SEM	Antimicrobial Activities	[84]

## Data Availability

Not applicable.

## Abbreviations

UV	Ultraviolet-visible spectroscopy
FESEM	Field Emission Scanning Electron Microscopy
XPS	X-Ray Photoelectron Spectroscopy
FTIR	Fourier-transform infrared spectroscopy
EDX	Energy Dispersive X-Ray Analysis
MTT	[3-(4.5- dimethylthiazol-2yl)]-2.5 diphenyl tetrazolium bromide
ROS	Reactive oxygen species
TEM	Transmission electron microscopy
TGA	Thermogravimetric Analysis
FE-TEM	Field emission transmission electron microscope
XRD	X-ray diffraction
Z-P	Zeta potential
DLS	Dynamic light scattering
ZnO NPs	Zinc oxide nanoparticles
